# Acute Anterior Choroidal Artery Territory Infarction: A Case Series Report

**DOI:** 10.3390/neurolint16020020

**Published:** 2024-02-29

**Authors:** Antonia Tsika, Polyxeni Stamati, Zisis Tsouris, Antonios Provatas, Alexandra Papa, Dimitrios Tsimoulis, Stylliani Ralli, Vasileios Siokas, Efthimios Dardiotis

**Affiliations:** Department of Neurology, University Hospital of Larissa, School of Medicine, University of Thessaly, 41100 Larissa, Greece; antonellatsi@hotmail.com (A.T.); tzeni_0@yahoo.gr (P.S.); tsouriszisis@me.com (Z.T.); antoinepr2004@hotmail.com (A.P.); papaalek@gmail.com (A.P.); tsimoulisdimitris@hotmail.com (D.T.); st_ralli@hotmail.com (S.R.); bill_s1983@hotmail.com (V.S.)

**Keywords:** anterior choroidal artery, anterior choroidal artery stroke, anterior choroidal artery infraction, stroke progression

## Abstract

Due to the occlusion of the anterior choroidal artery (AChA), ischemic strokes are described with the classic clinical triad, namely hemiplegia, hemianesthesia, and homonymous hemianopsia. The aim of this study is to document the characteristic clinical presentation and course of AChA infract cases. We describe five cases with acute infarction in the distribution of the AChA, admitted to the Neurological Department of the University General Hospital of Larissa. Results: All cases presented with hemiparesis and lower facial nerve palsy, while four of them had dysarthria, and two patients exhibited ataxia. Two cases underwent intravenous thrombolysis. A notable feature was the worsening of the clinical course, specifically the exacerbation of upper limb weakness within 48 h. Stabilization occurred after the third day, with the final development of a more severe clinical presentation than the initial one. Additionally, muscle weakness was more severe in the upper limb than in the lower limb. The recovery of upper limb function was poor in the three-month follow-up for the four cases. While vascular brain episodes are characterized by sudden onset, in AChA infraction, the clinical onset can be gradually developed over a few days, with a greater burden on the upper limb and poorer recovery.

## 1. Introduction

The anterior choroidal artery (AChA) is a small brain artery and represents the most distal branch of the internal carotid artery (ICA) and initiates immediately after the beginning of the posterior communicating artery (Pcomm) [1]. Its location is the main reason why occlusion in this territory is quite uncommon. It supplies blood to the anterior medial temporal lobe (the uncus, piriform cortex, amygdala, and head of the hippocampus), the optic tract, and several parts of the basal ganglia like the medial globus pallidus, the medial third of the cerebral peduncle, the geniculate body, portions of the ventral and pulvinar thalamus, and the posterior limb of the internal capsule [2]. Due to its blood supply to several brain structures, its occlusion, whether total or partial, can lead to variable clinical presentation. The most prevalent manifestations include pure motor symptoms with hemiparesis or hemiplegia, pure sensory symptoms affecting either the left or right side of the body, or even neglect syndrome, and both motor and sensory symptoms. Additional commonly observed symptoms are dysarthria, lower facial nerve palsy, and visual field disturbances such as homonymous hemianopia or scotoma, and quadrantic defects. Few studies have been conducted on infarctions in the distribution of the AChA, and the mechanism of clinical progression and prognosis is still unclear [3].

## 2. Materials and Methods

Our study describes five patients with acute stroke in the distribution area of the anterior choroidal artery, treated between 2021 and 2023 in the Neurological Department of the University General Hospital of Larissa.

## 3. Results

Case series.

### 3.1. CASE 1

**Figure 1:**
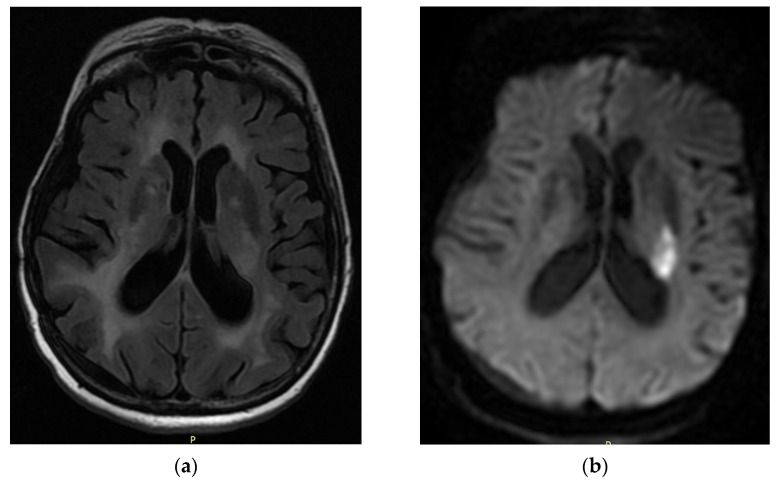
Brain MRI: (**a**) axial flair and (**b**) DWI, demonstrating acute ischemic infarction on left basal ganglia, in the distribution area of the left anterior choroidal artery.

The first case is a female, 53 years old, who presented with acute onset of dysarthria, dysmetria, lower facial weakness of the right side (VIIth cranial nerve), and right hemiparesis (4/5 muscle strength on the right arm and leg) (National Institutes of Health Stroke Scale (NIHSS: 5)). Her medical history consisted of surgically treated neuroblastoma with left pyramidal spastic hemiparesis, thyroidectomy, without any reported vascular risk factor, and was on levetiracetam 1000 mg and thyrohormone 0.1 mg. She was not a smoker or an alcohol abuser. She had no family history of cerebrovascular incidents. In the patient, antecedent neurological symptomatology on the right side was absent. The previous Modified Rankin Scale (pmRS) for neurologic disability was 2. The emergency brain CT showed chronic subdural hematomas in the curvature of the right hemisphere, right temporal lobe glial area, and left frontoparietal calcified meningioma. The brain MRI revealed an infarction in the distribution of the left anterior choroidal artery (Figure 1). On the following day of the admission, the clinical presentation of the patient worsened, leading to the right upper limb hemiplegia, without any motor worsening of the right lower limb (NIHSS: 7). During the next 48 h, there was instability concerning the patient’s clinical presentation, primarily related to muscular strength in the left upper limb (1/5 muscle strength) (NIHSS: 5–7). The new brain CT revealed no alterations. In subsequent days, no change in her clinical condition was observed. The ultrasound of the extracranial carotid arteries did not reveal hemodynamic stenoses. The echocardiogram was normal, and no episodes of atrial fibrillation were recorded on the 24 h rhythm Holter monitor. During hospitalization, high blood pressure was observed. The patient was discharged with antiplatelet treatment ASA (aspirin 100 mg), low-molecular-weight heparin (LMWH) for venous thromboembolism intervention, antihypertensive (amlodipine 5 mg), and statin (rosuvastatin 40 mg). During discharge, the National Institutes of Health Stroke Scale (NIHSS) score was 7, and the Modified Rankin Scale (mRS) for neurologic disability was 4. She was admitted to a rehabilitation center where she received intensive physiotherapy. Three months later, there was a slight improvement in the muscle weakness of the right upper limb, with a reduction in the remaining symptoms (NIHSS: 5, mRS: 3).

### 3.2. CASE 2

**Figure 2:**
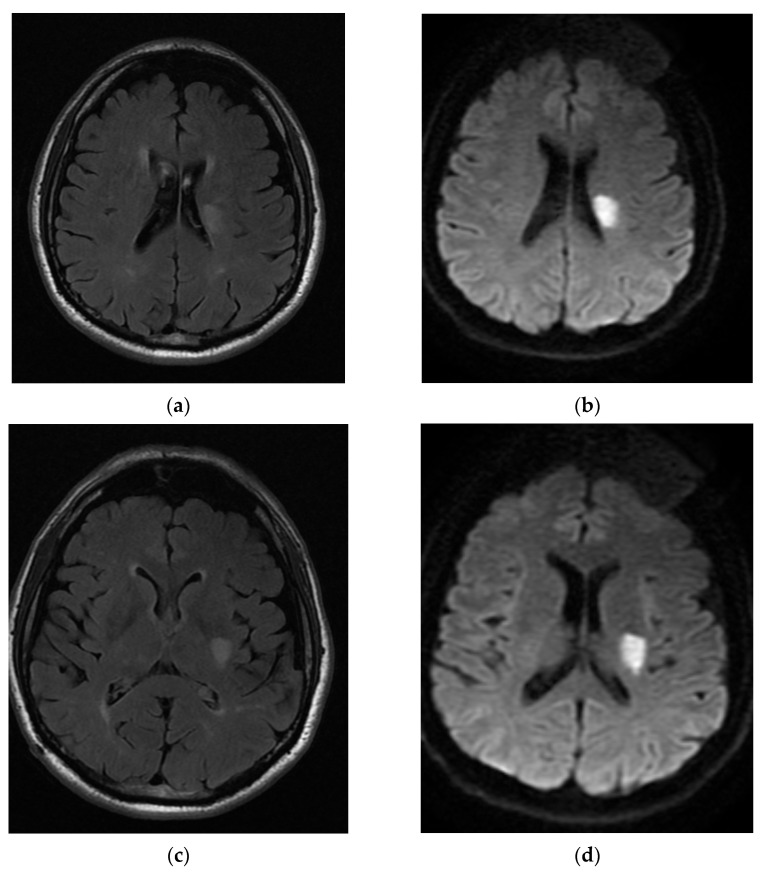
The patient’s brain MRI: axial flair (**a**,**c**) and DWI (**b**,**d**), with an acute ischemic lesion involving the left anterior choroidal artery. Also shown is ischemic microangiopathy.

A 52-year-old man was admitted with right hemiparesis (strength on arm 3/5 and on leg 4+/5) and minor facial weakness (NIHSS: 4). The patient reported current smoking and a history of arterial hypertension while he was on olmesartan 10 mg, and he had no family history of neurovascular events. The pmRS was 0, and the admission brain CT did not depict any hemorrhage or acute ischemic event. The patient received intravenous thrombolysis (IVT) with alteplase but without any improvement in the clinical status. The next day, the aggravation of hemiparesis (strength on arm 2/5 and on leg 4/5) (NIHSS: remained 4) occurred. The MRI scan is demonstrated in Figure 2, which reveals acute ischemic stroke in the distribution area of the left anterior choroidal artery and ischemic microangiopathy lesions. As shown in the imaging of the intracranial–extracranial vessels with CT angiography, there was no significant stenosis or atherosclerotic plaques. The transthoracic echocardiograph revealed mild left ventricular hypertrophy and the rhythm Holter monitor did not show any episodes of atrial fibrillation. Therefore, the comprehensive examination to ascertain the potential cause of the stroke revealed uncontrolled arterial blood pressure in the patient as the most probable etiology. During the discharge, the NIHSS was 3, the mRS was 3, and the prescribed treatment included antiplatelets (aspirin 100 mg), statin (atorvastatin 40 mg), folic acid, and the antihypertensive treatment he had already received. After 90 days, the mRS remained at 3.

### 3.3. CASE 3

**Figure 3:**
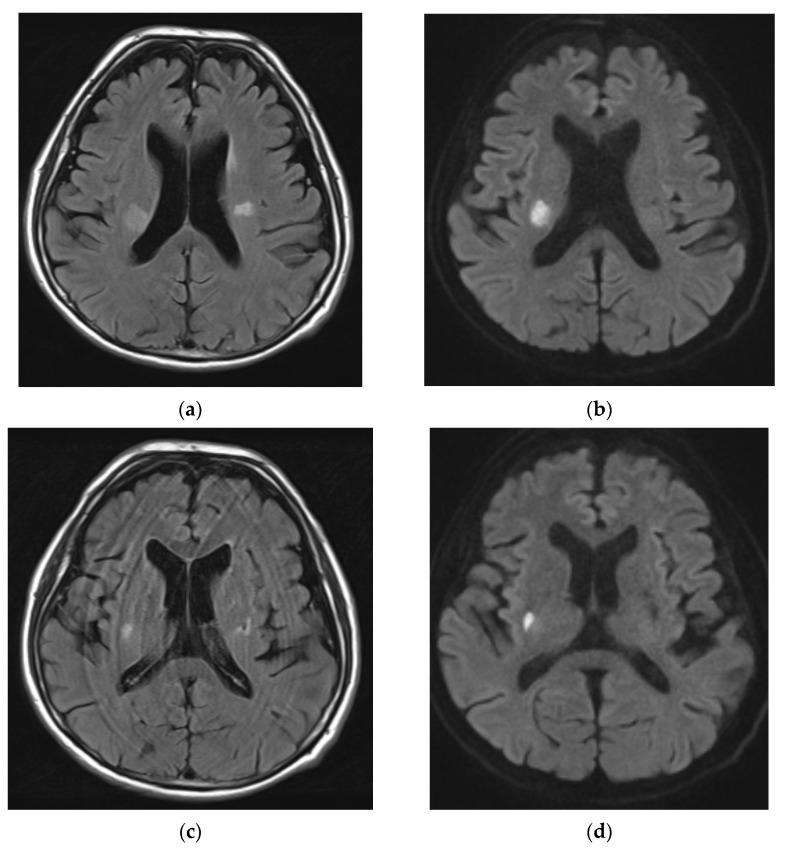
Brain MRI: axial flair (**a**,**c**) and DWI (**b**,**d**). This demonstrates the acute cerebral ischemia on the right AChA territory and an old ischemic stroke on the left AChA territory.

The fourth case pertains to a 58-year-old male presenting with several risk factors for cerebrovascular events, including arterial hypertension, dyslipidemia, and a history of smoking, without any family history. The patient was on clopidogrel 75 mg but was not receiving any medication for hypertension or dyslipidemia. The pmRS was 0. The patient manifested abrupt onset dysarthria and left-sided hemiparesis (muscle strength 4/5 on the arm and leg) (NIHSS: 3). The emergency brain CT revealed a neurovascular incidence on the left side of the lateral ventricle, which was not consistent with the clinical presentation. Antiplatelet treatment and statin started immediately. Over the course of 48 h, the clinical picture exhibited fluctuations, with periods of improvement and the subsequent exacerbation of muscle weakness on the contralateral side (NIHSS: 3–6). The ultimate clinical presentation proved more severe than the initial one upon admission, involving both right upper and lower limb weakness (NIHSS: 6). The brain MRI confirmed the AChA territory occlusion (Figure 3). Upon investigating the potential etiology of the ischemic event, pathological findings emerged in the color Doppler ultrasound examination of extracranial arteries, indicating mild atherosclerotic changes in the right carotid artery without significant stenosis, which was in accordance with the CT angiography of the extracranial and the intracranial vessel of the brain. The echocardiogram revealed left ventricular hypertrophy, likely attributable to arterial hypertension. Additionally, the rhythm Holter monitor recorded a nodal rhythm with branch block, devoid of episodes of atrial fibrillation. The episode was predominantly ascribed to an atherothrombotic event within the context of multiple risk factors. Upon discharge, the NIHSS was 6, and the mRS was 5. The prescribed medication included aspirin 325 mg, rosuvastatin 40 mg, and folic acid. During the 3-month follow-up, severe weakness persisted in the upper limb. However, the patient demonstrated the ability to stand, and dysarthria had been completely resolved (mRS: 3).

### 3.4. CASE 4

**Figure 4:**
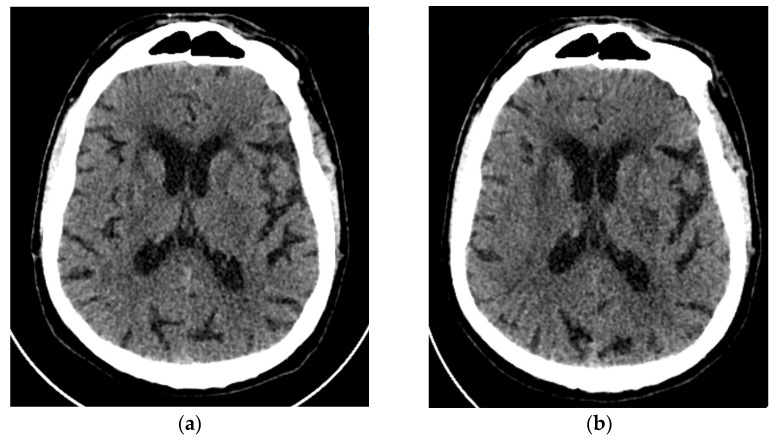
Axial brain CT scan (**a**) on an emergency basis with ischemic microangiopathy, without any obvious acute ischemic stroke or hemorrhage; (**b**) an axial CT scan 2 days later with a hypodense lesion in the left basal ganglia region, on the distribution of the left anterior choroidal artery (AChA) and ischemic microangiopathy periventricular and on the basal ganglia.

The subsequent case involves a male patient of advanced age, 77 years old, who presented with abrupt onset dysarthria and right-sided lower facial nerve paresis (NIHSS: 3). Notably, this individual had a medical history of coronary artery disease, type 2 diabetes mellitus, arterial hypertension, dyslipidemia, hepatic steatosis, myoclonus, asthma, and prostatic hyperplasia. He was on dual antiplatelet treatment/DAPT (clopidogrel 75 mg/aspirin 100 mg), brivaracetam 100 mg, ranolazine, irbesartan 300 mg/hydrochlorothiazide 25 mg, lansoprazole 30 mg, rosuvastatin 10 mg, metformin 850 mg, ursodeoxycholic acid, silodosin 8 mg, and dutasteride 0.5 mg. No family history was reported. The pmRS was also 0. The emergency brain CT did not reveal hemorrhage or acute cerebrovascular incident, and the DAPT was stopped with the consensus of the cardiologists as the acute coronary incidence was 6 years ago. Aspirin 325 mg was prescribed as antiplatelet treatment, and low-molecular-weight heparin (LMWH) was started for venous thromboembolism intervention. The axial brain CT, compared to the admission CT, revealed a hypodense lesion in the left basal ganglia region, correlating with the distribution of the anterior choroidal artery (AChA) (Figure 4). Subsequent CT angiography unveiled multiple atherosclerotic alterations, featuring stenoses of up to 50%, whereas notably asymptomatic and calcified atheromatous lesions were observed in the intracranial part of the internal carotids. The cardiological examination revealed mild left atrial dilation and mitral valve regurgitation, with the rhythm Holter monitor recording an absence of pathological arrhythmias. No brain MRI was performed during hospitalization since the 3Tesla MRI in our hospital was not suitable for this patient because of heart stenting. A follow-up CT scan revealed a more accentuated acute infarction in the left basal ganglia region, which confirmed the stroke. Throughout the initial 24 h period of hospitalization, the patient exhibited a deterioration in the clinical presentation, manifesting as hemiplegia of the right upper limb and muscular weakness in the right lower limb (3/5), persisting until the day of discharge (NIHSS: 7, mRS: 4). Upon reassessment in three months’ time, the patient showed clinical improvement in terms of dysarthria, although severe weakness persisted in the upper limb (mRS: 4).

### 3.5. CASE 5

**Figure 5:**
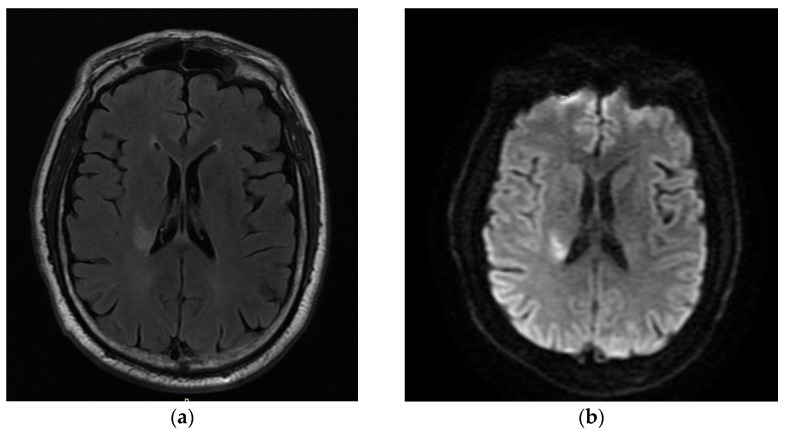
Brain MRI: axial flair (**a**) and DWI (**b**) manifesting right infarct extending from the right thalamus (posterior limb of the internal capsule) in the distribution of the right AChA.

The final case concerns a 54-year-old male with a medical history of type 2 diabetes mellitus, on dapagliflozin 10 mg and dulaglutide, untreated arterial hypertension, obstructive sleep apnea, smoking, and HIV infection contained with antiretroviral therapy (emtricitabine/tenofovir and darunavir/cobicistat). He had no family history of strokes. The pmRS was 0. The patient presented with abrupt onset ataxic hemiparesis (muscle strength 4/5 on the arm and leg) and dysarthria (NIHSS: 10), while the brain CT on admission showed no hemorrhage or acute/prior ischemic stroke; thus, thrombolysis was promptly provided within the established therapeutic window. After thrombolysis, there was remarkable clinical amelioration, with the persistence of a mild degree hemiparesis for a duration of two hours (NIHSS: 2). The patient received antiplatelet (aspirin 100 mg), antihypertensive (amlodipine 5 mg) treatment and statin (rosuvastatin 40 mg). However, a subsequent notable fluctuation in neurological symptoms ensued, ultimately becoming stabilized over the following 24 h with mild ataxic hemiparesis, predominantly affecting the upper limb (muscle strength 3/5 on the arm and 4/5 on the leg) (NIHSS: 3). Considering the variability observed and the concurrent HIV infection, a lumbar puncture was conducted, successfully excluding meningoencephalitis. Neuroimaging through brain MRI revealed an infarct extending from the right thalamus (the posterior limb of the internal capsule) to the right lateral ventricle, suggestive of anterior choroidal artery occlusion (Figure 5). Computed tomography angiography demonstrated atherosclerotic changes without significant stenosis. Atrial fibrillation was not detected during hospitalization. Further investigations for potential vasculitis yielded negative results. The patient was discharged with residual mild left hemiparesis and the treatment he received during hospitalization (NIHSS: 2, mRS: 2). The mRS at the three-month follow-up remained at 2.

## 4. Discussion

Strokes in the distribution area of AChA account for 2.9% to 11% of all ischemic strokes. The AChA syndrome is often characterized by hemiparesis attributed to internal capsule stroke, hemisensory loss from the damage of the thalamus or thalamocortical fibers, and hemianopia mainly due to involvement of the lateral geniculate body [4]. This is the classic triad of the complete occlusion of AChA. Hemianopia, a significant characteristic of the classic triad of AChA infarction, was not observed in any of our cases. All patients were presented with motor symptoms, aligning with the literature, which reports a frequency of 92.2%. However, none exhibited sensory symptoms, despite being the second most common manifestation (43%) [3,5,6]. Other reported symptoms included aphasia and the alteration of consciousness level, although cortical symptoms were exceedingly rare. One in ten individuals also presents symptoms from the posterior circulation system, such as dizziness and nausea, potentially attributed to the involvement of the thalamus and hippocampus [3]. All cases presented with hemiparesis and lower facial nerve palsy, while four of them also had dysarthria, and two patients exhibited ataxia (Table 1). None of our patients manifested the entirety of the classical triad. This stands as the predominant clinical presentation, given the infrequency of encountering occlusions that encompass the entire perfusion territory of the anterior choroidal artery due to its rich anastomoses with the other large arteries of this territory. Additionally, none of the patients demonstrated any impairment in cognitive or executive functions. Another noteworthy characteristic is the increased burden on the upper limb, exhibiting substantial asymmetry compared to the lower limb, an aspect that is not prominently highlighted in the international literature.

A distinctive feature of vascular cerebral events is the abrupt onset of symptoms. Although we presented a small case series, a distinctive characteristic was observed in all cases regarding the symptomatology. Despite the sudden onset, subsequent clinical presentation fluctuated within the initial 48 h, leading to the establishment of a more severe clinical picture than the initial one. This has also been reported by Ois et al., who stated that patients with AChA infarcts had lower mortality and risk of recurrence than patients with hemispheric lesions but a higher risk of clinical progression during the first few days after infarct [7]. It is possible for one to locate articles in the international literature that explicitly indicate the significance of the progressive manifestation of neurological symptoms in these cerebral cases [8]. It is also notable that, despite the absence of extensive infarctions and the acuteness of ischemic onset, there was an observed variability in the clinical presentation, prompting considerations. This variability raises concerns both in terms of patient management (potential cerebral expansion, hemorrhagic transformation, susceptibility to subarachnoid, and subdural bleeding) and in terms of the diagnosis of the stroke itself. It can be stated that a complete clinical and imaging profile of the AChA may not be deemed obligatory for discerning this progression.

The size of the infarcts varied, correlating with the severity of symptoms. No significant symptomatic atherothrombotic changes were found in the major vessels, and no atrial fibrillation was detected. Due to the small size of this artery, the infarction and fluctuation could be attributed to hemodynamic insufficiency, but neither previously published works nor our cases revealed significant hemodynamic instability or large fluctuations in arterial pressure [8]. Hypertension emerged as the most significant risk factor in most of our patients, which is documented in numerous studies as well [9,10] (Table 2). The mechanisms of small and large vessel disease overlap. Lacunar syndrome was present in 83.3% of patients, large-vessel disease was present in 38.1%, and cryptogenic disease in 38.1% according to the literature [11]. Two of our cases could be classified as atherothrombotic based on the investigation conducted to identify the etiology of the stroke, while in the remaining three cases, no clear pathophysiological mechanism was identified. The atherosclerosis of the carotid artery induced by hypertension, diabetes mellitus, and dyslipidemia appears to be closely associated with infarctions in the AChA [3].

Thrombolysis was performed in two out of the five cases, with one patient initially exhibiting favorable outcomes, characterized by a significant reduction in the NIHSS. Conversely, the second patient showed no response to thrombolysis, as the NIHSS remained stable both before and after the procedure. A few hours later, deterioration occurred without a change in radiological imaging. In accordance with Cheng’s findings, thrombolysis does not induce alterations in clinical presentation or prognosis at the 6-month time point. Nonetheless, the available data from studies are not substantial enough to derive definitive conclusions.

The recovery of upper limb function was poor in the three-month follow-up for four cases. MR tractography revealed that the corticospinal tract is involved in motor deficits and poor outcomes [5]. As in our cases, Derflinger et al. reported a 31% rate of mRS < 2 at hospital discharge, increasing to 89% at 3 months. However, not all studies align with this prognosis, as some associate outcomes with clinical progression, infarct size, and the presence of atrial fibrillation [3,8] (Table 3).

Our study had several limitations. We have a small number of cases, which does not allow us to draw any definite conclusion. We only describe our observations about these strokes.

## 5. Conclusions

It is crucial to consider the possible involvement of the AChA region in cases displaying significant symptom fluctuation during the initial hours post-stroke. Muscle weakness disproportionately greater in the upper limb compared to the lower limb should raise suspicion for the stroke localization in AChA region. Encouragingly, it appears that patients show improvement over time. Despite the initial concerns due to significant fluctuations, patients should be reassured, although the prognosis of upper limb weakness may not be as favorable. Most patients exhibited classic risk factors for stroke, emphasizing the importance of thorough investigation in these aspects. In conclusion, further studies with larger patient cohorts are necessary to elucidate the pathogenesis of the clinical evolution of these strokes, their precise etiology, and prognosis.

## Figures and Tables

**Table 1:** The clinical presentation of each of the 6 cases. All of them were presented with hemiparesis, and dysarthria was a common characteristic. None of the cases had the classic triad of AChA infarction (hemiparesis, hemisensory loss, and hemianopsia).

Clinical Presentation	Case 1	Case 2	Case 3	Case 4	Case 5
Dysarthria	+	-	+	+	+
VII central paresis	+	+	-	-	-
Hemiparesis	+	+	+	+	+
Hemisensory loss	-	-	-	-	-
Hemianopsia	-	-	-	-	-
Ataxia	+	-	-	-	+

**Table 2:** Previous known history of the 6 cases. Two cases did not have any known risk factor for stroke, but upon investigating the cause of the stroke, it was found that all patients had at least arterial hypertension.

Medical history	Case 1	Case 2	Case 3	Case 4	Case 5
Hypertension	-	+	+	+	+
Diabetes	-	-	-	+	+
Dyslipidemia	-	-	+	+	-
Smoking	-	+	+	-	+

**Table 3:** The NIHSS of each case, the 90-day mRS, and the main cause of the stroke according to the TOAST (Trial of Org 10172 in Acute Stroke Treatment) classification. Most of the patients were in their fifties and males. The mean NIHSS was 4–5, and the mRS was 3. Two patients had a lacunar stroke, two had large artery atherosclerosis, and the other two had ESUS (cryptogenic stroke and embolic stroke of undetermined source). Usually, strokes attributed to AChA occlusion are observed in hypertensive patients, although they have a larger diameter than lacunar strokes.

	Case 1	Case 2	Case 3	Case 4	Case 5
Sex	F	M	M	M	M
Age	53	52	58	77	54
NIHSS	5–7	4	3–6	3–7	2–10
Thrombolysis	-	+	-	-	-
Follow-up mRS	3	3	3	4	2
TOAST	ESUS	ESUS	LAA	LAA	ESUS

F: female, M: male, TOAST: Trial of Org 10172 in Acute Stroke Treatment, SVD: small-vessel disease, LAA: large artery atherosclerosis, ESUS: cryptogenic stroke and embolic stroke of undetermined source.

## Data Availability

The data presented in this study are available on reasonable request from the corresponding author.
